# The corneal thickness profile in different forms of glaucoma

**DOI:** 10.1038/s41598-025-26680-z

**Published:** 2025-11-07

**Authors:** Lisa Ramm, Wienke Fleischer, Robert Herber, Lutz E. Pillunat, Karin R. Pillunat

**Affiliations:** 1 Department of Ophthalmology, University Hospital Carl Gustav Carus, TU Dresden, Fetscherstr. 74, 01307 Dresden, Germany; 2 Ophthalmological Practice Dr. Cornelia Scheiber, Leipzig, Germany; 3 Faculty of Medicine Carl Gustav Carus, Department of Ophthalmology, TU Dresden, Dresden, Germany

**Keywords:** Glaucoma, Pachymetry, Corneal thickness profile, Corvis ST, Pentacam HR, Heidelberg Retina Tomograph, Perimetry, Eye diseases, Corneal diseases, Optic nerve diseases

## Abstract

To characterize the corneal thickness profile in primary high pressure (HPG) and normal pressure (NPG) open-angle glaucoma as well as pseudoexfoliative glaucoma (PEXG), and to analyze the influence of possible causative factors. Glaucoma patients and healthy age-matched subjects were included. In glaucoma, disease duration, current medication, functional (Humphrey perimetry) and structural (Heidelberg retina tomography) damage, and endothelial cell density (ECD) were assessed. The spatially resolved corneal thickness was measured with Pentacam HR. Thinnest corneal thickness (TCT) and peripheral pachymetry of concentric circles around TCT were determined. The Scheimpflug Analyzer Corvis ST was used to measure central corneal thickness (CCT) and pachy slope, which is an indicator of the change in corneal thickness from the apex to the periphery. After age adjustment, 112 healthy subjects and 286 patients (161 HPG, 76 NPG, 49 PEXG) were included. In glaucoma, CCT, TCT and pachy slope were significantly reduced (P < 0.001). Likewise, peripheral pachymetry at various distances (P < 0.001) and the differences between TCT and periphery (P < 0.019) were lower than in healthy subjects. There were also significant deviations when analyzing the different forms of glaucoma (all group comparisons P < 0.001, with lower pachymetric values in each patient group compared to healthy controls). Furthermore, the changes were associated with parameters of structural damage (e.g. pachy slope and rim area, P < 0.001), disease duration (PAC 5 mm, P = 0.016) and use of prostaglandin analogues (e.g. CCT, pachy slope, PAC 5 mm, all P ≤ 0.05). Glaucoma involves corneal thinning, affecting not only the center but also the periphery. The physiological increase in peripheral corneal thickness was reduced in all glaucoma subgroups. The associations with influencing factors might indicate pathogenetic mechanisms.

**Supplementary Information:**

The online version contains supplementary material available at 10.1038/s41598-025-26680-z.

## Introduction

Glaucoma is one of the most common causes of blindness worldwide. The global prevalence among 40 to 80 year olds is estimated at 3.5%. By 2040, around 111.8 million people are expected to become ill^1^. Although the great relevance of the disease is beyond question, the pathogenesis is not clear and lowering intraocular pressure (IOP) is the only evidence-based therapeutic approach^2,3^. There is a great interest in clarifying the causal pathogenetic mechanisms and thereby opening up the opportunity for new and efficient therapeutic approaches.

Aspects discussed so far in the pathogenesis of glaucomatous optic neuropathy include genetic factors, disorders of axonal transport and deficiency of trophic factors, autoimmunity, glutamate excitotoxicity and changes in electrical activity of cells^3,4^. In addition, the perfusion of the retina and the optic nerve head (ONH) could be disturbed. Impaired neurovascular coupling with vascular dysregulation, vasospasm and reduced perfusion, mechanical vascular compression and the formation of reactive oxygen species are discussed^5–7^. There are also indications of remodeling processes in the ocular tissue. Previous studies reported stiffness changes in the sclera, the lamina cribrosa and the trabecular meshwork^8–11^. Increased strength of lamina cribrosa and peripapillary sclera may raise the susceptibility of the ONH to glaucomatous injury due to intraocular pressure fluctuations^8,12^. In addition, chronic remodeling of the ONH connective tissue is considered to be the cause of progressive glaucomatous cupping^13,14^. The anterior continuation of the ocular covers forms the cornea, which also appears to be subject to glaucomatous changes. Alterations in corneal biomechanical properties^15–17^ and changes in central corneal thickness (CCT)^18–20^ are known in glaucoma. The Ocular Hypertension Study showed, that thinner CCT is a risk factor for the development of primary open angle glaucoma (POAG) in the setting of existing ocular hypertension^21^. Consistently, the Early Manifest Glaucoma Trail identified a thinner CCT as progression factor in patients with higher IOP^22^, and the European Glaucoma Prevention Study also confirmed the CCT as a significant predictor of glaucoma^23^. The results comparing the absolute thickness of the central cornea between healthy subjects and those with glaucoma are inconsistent. In most cases, glaucomatous damage was associated with a reduction in CCT^18–20^.

However, so far only the central pachymetry of the cornea has been considered. Therefore, the aim of this work is to characterize corneal thinning more precisely, taking into account the peripheral corneal thickness profile. By examining possible associated factors and considering potential deviations between different forms of glaucoma, information about the underlying pathogenetic mechanisms of tissue changes should be collected.

## Methods

The study was conducted at the department of Ophthalmology, University Hospital Carl Gustav Carus, TU Dresden, Germany. The study protocol conformed to the principles of the Declaration of Helsinki and was approved by the local ethics committee (EK 433,102,015, NCT02959242). Informed consent was obtained from all study participants before inclusion. The data were collected between January 2016 and September 2018. Patients with glaucoma were admitted for a one-day in hospital routine glaucoma work up. The diagnosis for glaucoma was based on the presence of typical ONH changes with a reduction of the neuroretinal rim and glaucomatous cupping as well as characteristic visual field defects^2^. In all glaucoma patients, in addition to funduscopic evaluation, an objective examination of the ONH was performed using confocal scanning laser ophthalmoscopy with the Heidelberg Retina Tomograph (HRT II, Heidelberg Engineering Inc., Heidelberg, Germany). Furthermore, Bruch’s membrane opening (BMO) analysis, ganglion cell complex imaging, and retinal nerve fiber layer (RNFL) analysis were conducted in all patients using optical coherence tomography (Spectralis-System, Heidelberg Engineering Inc., Heidelberg, Germany) to ensure an accurate diagnosis. POAG was verified using gonioscopy. These patients were divided into a normal (NPG) and a hypertensive (HPG) group, with NPG being defined as POAG with a history of untreated IOP ≤ 21 mmHg. The diagnosis for pseudoexfoliative glaucoma (PEXG) was based on additional slit lamp microscopic detection of pseudoexfoliative material by an ophthalmologist. The IOP-lowering medication was not paused.

The results of the study measurements were compared with a group of age-matched healthy subjects. A manual iterative matching approach was applied, involving stepwise case exclusion to achieve mean balance.

Exclusion criteria for glaucoma patients and healthy subjects were any preexisting corneal disease, traumata or ocular surgery in the past (except for uncomplicated phacoemulsification at least 3 months prior to the study measurements), contact lens wear, systemic connective tissue diseases, and lacking capacity to consent.

An ophthalmological examination of the anterior segment and the fundus of the eye as well as an IOP measurement were performed. Glaucoma patients underwent 24-h IOP assessment using Goldmann applanation tonometry. Six discrete IOP measurements were obtained at defined time points (7 a.m., 1 p.m., 4 p.m., 7 p.m., 10 p.m., and 12 a.m.). The midnight measurement (12 a.m.) was performed after at least one hour in a supine position. The mean of these six readings was used for further analysis. Automated perimetry was performed using the Humphrey field analyzer (Swedish interactive threshold algorithm standard 30–2 program, Carl Zeiss Meditec., Dublin, CA, USA), and the indices of mean deviation (MD) and pattern standard deviation (PSD) were determined. As mentioned above, HRT was performed, and the parameter rim area was analyzed in glaucoma. The corneal endothelial cell density (ECD) was measured using a specular microscope (CME-530, Nidek Technologies, Albignasego, Italy). In addition, the patients´ duration of the disease and current medication were recorded. Only one eye of each participant was included in further analysis. If there were no exclusion criteria, the eye with the more advanced damage was selected.

Study measurements were performed using the Scheimpfluganalyzer Corvis ST (CST, Oculus, Wetzlar, Germany) and the Pentacam HR (type 70,900, Oculus, Wetzlar, Germany). Details of the CST have been published elsewhere^24,25^. In brief, the measurement is based on the applanation of the cornea. A rapid airflow causes the cornea to move inward until it reaches a concave state. The intensity of the air flow then decreases and the cornea returns to its original shape due to its own elasticity. During corneal deformation, a high-speed Scheimpflug camera records the corneal movement. Measurements of amplitude, duration and velocity of the induced applanation provide information about corneal biomechanical properties^24,25^. Furthermore, the spatially resolved corneal thickness can be measured from the two-dimensional image of the cross-section of the deforming cornea. The device provides the CCT and the parameter pachy slope. The pachy slope represents the difference between the corneal thickness at the apex and the peripheral average, which is determined at 2.5 mm from the apex. Thereby, the apex represents the most anterior corneal point^26^. The following formula is used to calculate pachy slope: (1) Pachy 1 [μm] = Pachy (Apex + 2.5 mm) [μm]; (2) Pachy 2 [μm] = Pachy (Apex−2.5 mm) [μm]; (3) Pachy slope [μm] = (Pachy 1 [μm] + Pachy 2 [μm])/2−Pachy (Apex) [μm]^26^. Measurements with the CST were only taken once in every eye since previous reports described reliable and good quality results even after a single measurement time point^27,28^. The Pentacam measures the spatially resolved corneal thickness and the characteristics of the anterior segment of the eye. It uses a rotating Scheimpflug camera and provides two-dimensional optical sections during a 180-degree rotation. From the images, the device reconstructs the three-dimensional anterior and posterior surface topography of the cornea. The distance between the anterior and the posterior border of the cornea at each point represents the spatially resolved corneal thickness. Peripheral pachymetry was estimated as follows^26^: The software identifies the point of thinnest corneal thickness (TCT) and determines the mean corneal thickness value on concentric rings around TCT. Since the device provides data from rings in 0.4 mm increments, the values for circles with diameters of 2.5 mm, 5 mm and 7.5 mm (corresponding to PAC 2.5 mm, PAC 5 mm, PAC 7.5 mm; PAC: average peripheral pachymetry value of concentric rings around TCT) were calculated by linear interpolation (averaging the two nearest neighboring rings) in µm. Furthermore, to represent the change in corneal thickness from the center to the periphery, the differences between the TCT and the concentric peripheral rings around it, were calculated (corresponds to PAC_Diff_ 2.5 mm, PAC_Diff_ 5 mm, PAC_Diff_ 7.5 mm). The CST parameter pachy slope is approximately equal to the difference between the TCT and the 5 mm diameter value (PAC_Diff_ 5 mm). However, the values cannot be equated because the Pentacam uses mean pachymetry values of concentric circles in relation to the TCT. On the other hand, the pachy slope represents the difference between the apex and two points located on a horizontal line. Repeatability data from the literature indicate high reliability for the Pentacam HR in both central and peripheral pachymetry^29–31^.

The sample size planning was carried out using the GPower 3.1.9.2 program (Heinrich Heine Universität Düsseldorf, Germany) based on the assumption of 80% power and an alpha error of 0.05. The measurement data from 15 healthy volunteers, 15 HPG and 15 NPG patients were used for analysis, which were subsequently not included in the study calculations.

The comparison of pachy slope between glaucoma and healthy subjects resulted in a necessary group size of 24 people each. The question about a possible difference in pachy slope between HPG and NPG resulted in a number of 33 subjects per group. To compare CCT between healthy people and patients, a sample size of 8 per group was necessary. To investigate a possible difference in CCT between HPG and NPG, a group size of 61 patients was calculated. For PAC 5 mm, 13 subjects per group were required to detect a difference between glaucoma patients and healthy subjects. To find a difference between HPG and NPG, 30 subjects per group were required.

Study data were analyzed using the software SPSS (Version 29, IBM Statistics, New York, USA). The primary endpoints were (1) pachy slope, (2) PAC 5 mm, and (3) CCT. Pachy slope represents the spatial gradient of corneal thickness, PAC 5 mm reflects the paracentral zone whose change was evaluated according to the study hypothesis, and CCT serves as a clinically established reference parameter. Secondary endpoints were defined as TCT and PAC_Diff_ 5 mm. The Parameters PAC 2.5 mm, PAC 7.5 mm, PAC_Diff_ 2.5 mm, and PAC_Diff_ 7.5 mm were analyzed exploratively. The Kolmogorov–Smirnov test was used to check for normal distribution. Depending on the outcome, the Student´s t-test or the Mann–Whitney-U test was applied for simple group comparisons, and the Chi-square test was utilized for nominal data. To compare healthy subjects and patients with different forms of glaucoma, ANOVA with Bonferroni correction or Kruskal–Wallis test was used. Standardized effect sizes were calculated as η^2^ for ANOVA and rank-biserial r for Kruskal–Wallis tests to enable comparison across glaucoma subtypes. For the primary outcome parameters CCT, pachy slope and PAC 5 mm, the associations between the corneal parameters and possible influencing factors were examined using Spearman or Pearson correlation analysis. To correct for confounders, multivariable linear models were calculated for the main corneal outcome measures. Covariates included age, sex, lens status, glaucoma subtype, disease duration, IOP, number of medications, local prostaglandin analogues (PGA) application and ECD. All categorical predictors were modeled as indicator variables in the multivariable linear regression analysis. Since the ECD was only available for 137 of 286 glaucoma patients, two models were calculated (for the subgroup with available values, model A and model B). The reason for the missing values was that this measurement was performed only in a subset of patients (randomly selected), and the measurement data were not automatically stored by the examination device. To control for multiple testing, in addition to the Bonferroni correction for glaucoma subgroup comparisons, the false discovery rate (FDR) was controlled using the Benjamini–Hochberg procedure, which was applied separately to all groups of related tests (all correlation analyses (n = 18) and all pairwise group comparisons for pachymetry metrics (n = 21)). Adjusted p-values (p_FDR_) are presented next to the original p-values, and significance was set at p_FDR_ < 0.05.

## Results

Data from a total of 286 glaucoma patients and 112 age-matched healthy subjects were included in the analysis. According to age matching, there was no significant age difference between the groups (P = 0.875). Baseline data of the participants are given in Table 1.

**Table 1:** Baseline data and comparison of corneal parameters between healthy subjects and glaucoma patients (P-value: comparison between healthy subjects and glaucoma patients overall using: ^1^ T-Test, ^2^ Mann–Whitney-U-Test, ^3^ Chi^2^-Test; mean value and standard deviation, median and interquartile range 25/75; HPG: high pressure primary open angle glaucoma, NPG: normal pressure primary open angle glaucoma, PEXG: pseudoexfoliative glaucoma, IOP: intraocular pressure, ECD: endothelial cell density, MD: mean deviation, PSD: pattern standard deviation, PGA: prostaglandin analogues, CAI: carbonic anhydrase inhibitors).

	Healthy subjects	Glaucoma (overall)	P	HPG	NPG	PEXG
Baseline data						
Number	n = 112	n = 286	-	n = 161	n = 76	n = 49
Age (years)	70.4 ± 8.3 70 (63; 78)	69.7 ± 9.7 72 (63; 77)	0.875^2^	69.5 ± 9.4 71 (62; 77)	67.9 ± 10.3 70.5 (62.3; 76)	72.9 ± 8.9 75 (67.5; 79.5)
Male/female	59/53	125/161	0.106^3^	74/87	27/49	24/25
Right/left eye	65/47	142/144	0.132^3^	82/79	38/38	22/27
IOP (mmHg)	14.3 ± 3.1 14 (12; 16)	16.7 ± 5.9 15 (12,7; 19)	0.001^2^	17.3 ± 5.5 15.8 (13.7; 19.2)	12.7 ± 2 12.4 (11.2; 13.5)	21.2 ± 7.4 19.3 (15.8; 25.3)
ECD (/mm^2^)	2489 ± 137 2489 (2392; 2586)	2146 ± 465 2180 (1862; 2402)	0.3^1^	2137 ± 474 2149 (1851; 2480)	2335 ± 285 2385 (2202; 2591)	2094 ± 490 2164 (1806; 2351)
Pseudo-/phakic	2/110	85/201	< 0.001^3^	52/109	12/64	21/28
Perimetry and Heidelberg retina tomography						
MD (dB)	–	-10.9 ± 8.3 -8.6 (-16.2; -4.1)	–	-10.4 ± 8.1 -8.2 (-16.2; -3.6)	-8.7 ± 6.2 -6.9 (-12.6; -3.8)	-16.2 ± 9.7 -14.8 (-25.3; -8.1)
PSD (dB)	–	7.8 ± 4.1 8.1 (3.7; 11.1)	–	7.4 ± 4 7.5 (3.4; 11.1)	8.5 ± 4,5 8.6 (4.3; 12.3)	7.9 ± 3.4 8.3 (5; 10.9)
Rim area (mm^2^)	-	1.02 ± 0.42 1 (0.74; 1.27)	–	1.01 ± 0.39 0.99 (0.75; 1.21)	1.04 ± 0.37 1.06 (0.77; 1.33)	1.05 ± 0.58 0.92 (0.67; 1.36)
Antiglaucomatous local therapy						
Substances	-	3.2 ± 1.3 4 (2; 4)	–	3.4 ± 1.2 4 (3; 4)	2.6 ± 1.5 3 (1; 4)	3.6 ± 0.9 4 (3; 4)
PGA	-	255 out of 286 (89.2%)	–	148 out of 161 (91.9%)	62 out of 76 (81.6%)	45 out of 49 (91.8%)
CAI	-	221 out of 286 (77.3%)	–	127 out of 161 (78.9%)	50 out of 76 (65.8%)	44 out of 49 (89.8%)

With regard to IOP-lowering medications, the following proportions were found for all glaucoma patients: 75.9% beta-blockers (217 out of 286), 89.2% PGA (255 out of 286), 77.3% carbonic anhydrase inhibitors (CAI, 221 out of 286), 66.8% alpha-2-selective adrenergic agonists (191 out of 286) and 18.2% parasympathomimetics (52 out of 286). The frequencies of PGA and CAI use within the different glaucoma groups are shown in Table 1.

**Fig. 1:**
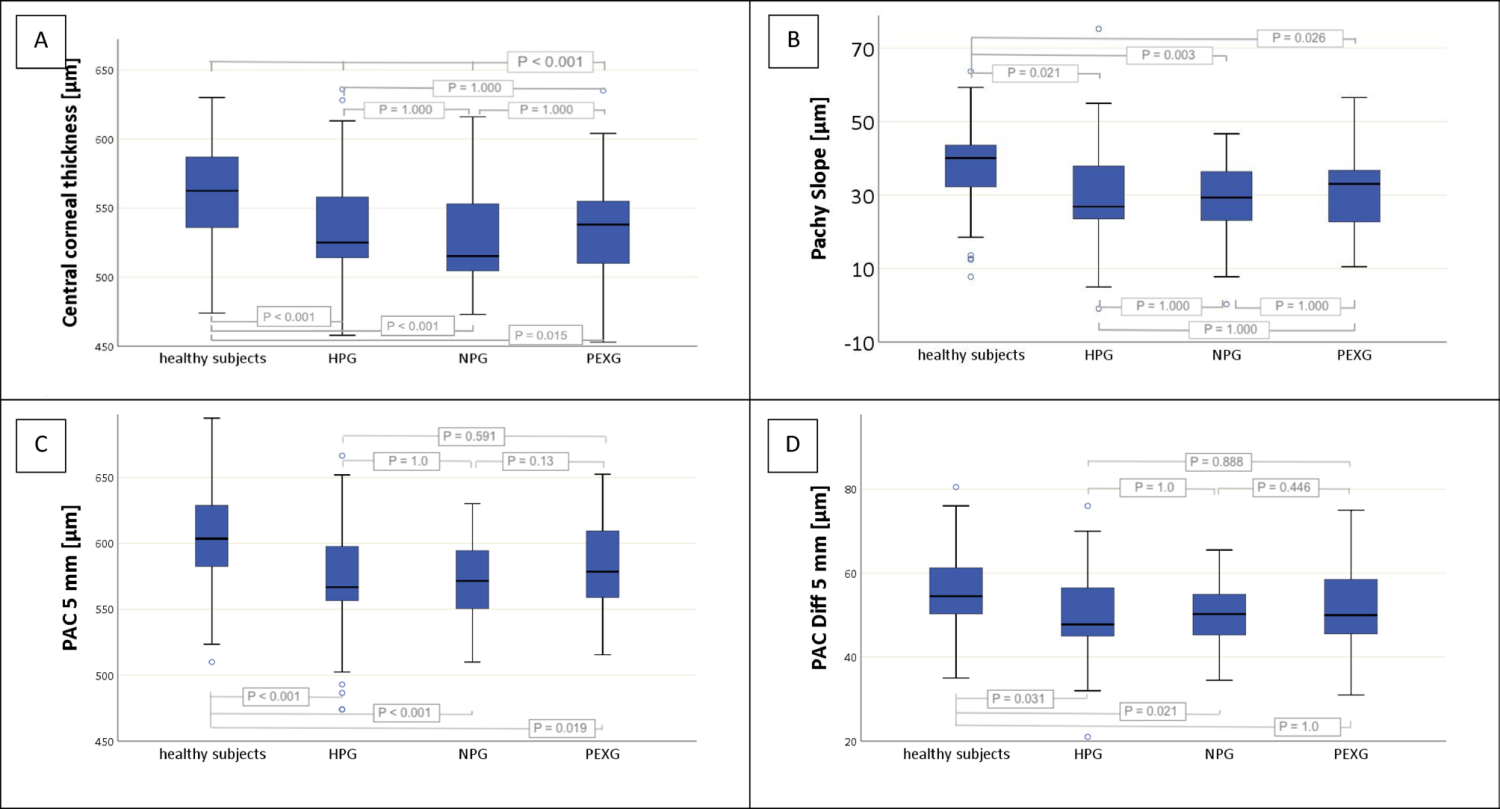
Comparison of corneal parameters between healthy subjects and patients with different forms of glaucoma (*HPG* High pressure primary open angle glaucoma, *NPG* Normal pressure primary open angle glaucoma, *PEXG* Pseudoexfoliative glaucoma, *PAC* 5 mm: average pachymetry value of the concentric ring around thinnest corneal thickness with a diameter of 5 mm, PAC_Diff_ 5 mm: difference in pachymetry values between thinnest corneal thickness and PAC 5 mm).

CST measurements revealed significantly lower CCT and pachy slope values in glaucoma compared to healthy subjects (P_FDR_ < 0.001). This was consistent with the spatially resolved pachymetry measurements using the Pentacam. The results of peripheral measurements at different distances from the center of the cornea (PAC) also showed highly significantly lower values in glaucoma patients than in healthy people (P_FDR_ < 0.001). The change in corneal thickness from center to the periphery (PAC_Diff_) was also lower in patients than in healthy subjects. This means that the physiological increase in thickness of the cornea from the center to the periphery is reduced in glaucoma patients. When the pachymetry values were compared between healthy subjects and the different forms of glaucoma, analogous results were obtained (results in Table 2 and corresponding effect sizes in the supplementary table). As an example, the results for CCT, pachy slope, PAC 5 mm and PAC_Diff_ 5 mm are illustrated in Fig. 1A-D. PAC_Diff_ 5 mm was not significantly different between PEXG and normal subjects (P = 1.0, Fig. 1D). All other individual group comparisons showed clear divergences from normal subjects.

**Table 2:** Comparison of corneal parameters between healthy subjects and glaucoma patients (left^Δ^: comparison between healthy subjects and glaucoma patients overall, mean difference and 95% confidence interval, original P-value using ^1^ T-Test or ^2^ Mann–Whitney-U-Test and adjusted P-value (P^**Δ**^_FDT_) using the Benjamini–Hochberg procedure; right^⋄^: subgroup analysis between healthy subjects and different forms of glaucoma, P-value using ^1^ ANOVA with Bonferroni correction or ^2^ Kruskal–Wallis-test; mean value and standard deviation, median and interquartile range 25/75; HPG: high pressure primary open angle glaucoma, NPG: normal pressure primary open angle glaucoma, PEXG: pseudoexfoliative glaucoma, CCT: central corneal thickness, TCT: thinnest corneal thickness, PAC: average peripheral pachymetry value of concentric rings around TCT (circle diameter indicated in mm), PAC_Diff_: difference of pachymetry values between TCT and concerning concentric ring; all values indicated in μm).

	Healthy subjects	Glaucoma (overall)	Mean differencs	P^Δ^	P^Δ^_FDT_	HPG	NPG	PEXG	P^⋄^
Scheimpfluganalyzer Corvis ST									
CCT	563 ± 37 563 (536; 587)	534 ± 37 534 (511; 556)	29 (-37.1; -20.9)	< 0.001^1^	< 0.001	536 ± 37 536 (514; 559)	530 ± 33 526 (504; 554)	536 ± 39 538 (509; 557)	< 0.001^1^
Pachy slope	37.4 ± 10.8 36.6 (32.1; 44)	30.5 ± 17.7 29.9 (23.2; 37.3)	-6.9 (-9.8; -4)	< 0.001^2^	< 0.001	31.6 ± 21.7 30.2 (23.4; 38)	28.9 ± 9.4 29.3 (22.8; 36.4)	29.5 ± 11.9 29.4 (21.4; 37)	< 0.001^2^
Pentacam HR									
TCT	549 ± 36 548 (529; 570)	526 ± 33 526 (505; 547)	-23 (-30.7; -15.3)	< 0.001^1^	< 0.001	526 ± 36 526 (505; 548)	521 ± 30 523 (504; 543)	532 ± 31 528 (511; 557)	< 0.001^1^
PAC 2,5 mm	565 ± 37 563 (544; 583)	540 ± 34 539 (519; 561)	-25 (-32.9; -17.1)	< 0.001^1^	< 0.001	540 ± 35 539 (519; 561)	535 ± 30 536 (515; 556)	549 ± 33 542 (525; 574)	< 0.001^1^
PAC 5 mm	605 ± 38 604 (583; 629)	577 ± 35 575 (555; 597)	-28 (-36.1; -19.9)	< 0.001^1^	< 0.001	577 ± 35 577 (555; 598)	571 ± 32 572 (550; 595)	587 ± 38 579 (559; 610)	< 0.001^1^
PAC 7,5 mm	666 ± 56 667 (642; 695)	630 ± 54 633 (609; 659)	-36 (-48.1; -23.9)	< 0.001^1^	< 0.001	631 ± 56 631 (609; 660)	628 ± 34 627 (603; 649)	629 ± 74 641 (612; 672)	< 0.001^2^
PAC_DIFF_ 2,5 mm	15 ± 3 15 (14; 17)	14 ± 7 14 (12; 16)	-1 (-2; 0)	< 0.001^2^	< 0.001	14 ± 4 14 (12; 16)	14 ± 2 13 (12; 16)	17 ± 15 14 (12; 17)	< 0.001^2^
PAC_DIFF_ 5 mm	56 ± 9 55 (50; 61)	51 ± 14 50 ( 45; 56)	-5 (-7.3; -2.7)	0.003^1^	0.008	51 ± 11 51 (45; 57)	50 ± 7 50 (45; 55)	54 ± 26 50 (45; 59)	< 0.001^2^
PAC_DIFF_ 7,5 mm	116 ± 35 118 (104; 129)	100 ± 61 107 (95; 120)	-16 (-25.6; -6.4)	0.008^1^	0.019	102 ± 53 107 (95; 122)	106 ± 15 108 (98; 117)	84 ± 110 107 (94; 122)	< 0.001^2^

Subsequently, the influence of possible causal factors was considered. Due to the number of parameters, the following analyses were performed only for the parameters CCT, pachy slope and PAC 5 mm in all glaucoma patients. First, it was examined whether there was an association with the severity of the glaucomatous damage. Only PSD and PAC 5 mm correlated significantly before adjustment (r = − 0.128; P = 0.032; P_FDR_ = 0.128). The rim area was associated to pachy slope (r = 0.197, P < 0.001; P_FDR_ < 0.001) and PAC 5 mm (r = 0.151, P = 0.011; P_FDR_ = 0.066). Furthermore, it was examined whether uneventful phacoemulsification in the past had an influence. The comparison of the parameters mentioned between phakic and pseudophakic patients revealed no significant difference in the corneal parameters in either healthy subjects or glaucoma patients (P_FDR_ > 0.05). Moreover, it was investigated whether the antiglaucomatous local therapy has an influence on the corneal thickness profile. The number of active substances used correlated with pachy slope in glaucoma only before adjustment (r = -0.118, P = 0.047; P_FDR_ = 0.11). Comparison of corneal parameters between glaucoma patients overall with and without CAI use revealed no significant differences. However, differences were found between patients with and without PGA use. When all glaucoma cases were included, the comparison of CCT (533 ± 36 vs. 550 ± 39 μm, P = 0.014, P_FDR_ = 0.029), pachy slope (30.1 ± 18.5 vs. 33.6 ± 9.4 μm, P = 0.029, P_FDR_ = 0.05), and PAC 5 mm (576 ± 35 vs. 590 ± 35 μm, P = 0.014, P_FDR_ = 0.027) showed significant differences between patients with and without PGA. To minimize potential bias due to differences in glaucoma severity between patients with and without PGA use, several indicators of disease severity were compared between the two groups (MD: -11.1 ± 8.2 vs. -9.7 ± 8.6 dB, PSD: 7.4 ± 4 vs. 6.9 ± 4.5; rim area: 1.02 ± 0.43 vs. 1.1 ± 0.35 mm^2^, mean RNFL thickness: 157 ± 187 vs. 157 ± 90 µm, disease duration 12.1 ± 9.8 vs. 12.5 ± 8.7 years). No significant differences were found (all p > 0.082). In order to rule out that the alterations found in corneal thickness profiles were only due to the influence of PGA, an additional analysis was carried out to compare the parameters of healthy subjects with the entire group of glaucoma patients without PGA use. A significant difference in the parameter pachy slope remained between healthy individuals and patients (38.4 ± 10.1 vs. 33.6 ± 9.4 μm, P = 0.039, P_FDR_ = 0.049). Finally, the possible influence of the ECD on corneal thickness profile in glaucoma was examined. A current ECD measurement was available for 137 patients (81 HPG, 15 NPG and 41 PEXG patients). It correlated with CCT in glaucoma patients only before control for multiplicity (r = 0.176, P = 0.039, P_FDR_ = 0.117).

Using multivariable linear regression analysis with adjustment for confounders, only significant associations were found between corneal parameters and glaucoma duration, IOP, and local application of PGA (see Table 3).

**Table 3:** Results of multivariable linear regression for primary corneal outcome parameters to adjust for confounding (The corneal parameters central corneal thickness (CCT), pachy slope, and average peripheral pachymetry value of concentric 5 mm ring around thinnest corneal thickness (PAC 5 mm)) were adjusted for age, sex, lens status, glaucoma subtype, disease duration, intraocular pressure (IOP), number of medications, use of prostaglandin analogues (PGA) and endothelial cell density (ECD). Adjusted regression coefficients (beta), 95% confidence intervals, and exact p-values are reported. Significant results are shown in bold. Since ECD was available in only 137 of 286 patients, a model with (left side, model A) and without (right side, model B) ECD was created.)

	CCT (Beta, 95% CI, p)	Pachy slope (Beta, 95% CI, p)	PAC 5 mm (Beta, 95% CI, p)	CCT (Beta, 95% CI, p)	Pachy slope (Beta, 95% CI, p)	PAC 5 mm (Beta, 95% CI, p)
Model A				Model B		
Age (years)	-0.03 (-0.833; 0.617) 0.769	-0.01 (-0.535; 0.487) 0.926	-0.071 (-1.029; 0.496) 0.49	0.006 (-0.414; 0.452) 0.931	-0.098 (-0.418; 0.055) 0.132	0.012 (-0.419; 0.51) 0.847
Male/female	-0.071 (-16.965; 7.428) 0.44	0.099 (-3.962; 13.219) 0.288	-0.022 (-14.342; 11.3) 0.815	-0.054 (-11.739; 4.46) 0.377	0.079 (-1.543; 7.306) 0.201	-0.024 (-10.416; 6.942) 0.694
Pseudo-/phakic	0.103 (-6.016; 19.806) 0.292	0.117 (-3.627; 14.562) 0.236	0.073 (-8.452; 18.693) 0.457	0.03 (-7.373; 11.716) 0.655	0.088 (-1.752; 8.674) 0.192	-0.021 (-11.831; 8.624) 0.758
Glaucoma subtype	-0.041 (-8.503; 5.386) 0.658	-0.123 (-8.186; 1.597) 0.185	-0.001 (-7.33; 7.271) 0.994	-0.012 (-5.992; 4.925) 0.847	-0.086 (-5.076; 0.887) 0.168	0.006 (-5.565; 6.133) 0.924
Disease duration (years)	**-0.198 (-1.154;** **-0.026) 0.041**	-0.095 (-0.596; 0.199) 0.325	**-0.203 (-1.227;** **-0.041) 0.036**	-0.122 (-0.818; 0.01) 0.056	-0.017 (-0.256; 0.196) 0.792	**-0.145 (-0.957;** **-0.07) 0.024**
IOP (mmHg)	0.087 (-0.483; 1.418) 0.332	**0.222 (0.159;** **1.498) 0.016**	0.043 (-0.755; 1.243) 0.629	**0.211 (0.51;** **1.889) 0.001**	**0.157 (0.103;** **0.856) 0.013**	**0.184 (0.374;** **1.852) 0.003**
Number of medications	0.148 (-2.732; 11.394) 0.227	0.038 (-4.207; 5.743) 0.76	0.132 (-3.391; 11.459) 0.284	0.03 (-3.312; 4.92) 0.701	-0.024 (-2.602; 1.895) 0.757	0.003 (-4.311; 4.511) 0.964
PGA exposure	**-0.27 (-59.41;** **-3.259) 0.029**	-0.055 (-24.213; 15.34) 0.658	-0.226 (-56.927; 2.102) 0.068	**-0.151 (-33.026; -0.816) 0.04**	-0.06 (-12.419; 5.174) 0.418	-0.127 (-32.422; 2.093) 0.085
ECD (/mm^2^)	0.125 (− 0.004; 0.023) 0.185	0.045 (− 0.007; 0.012) 0.635	0.125 (− 0.005; 0.024) 0.187	–	–	–

## Discussion

Consistent with most previous studies^18–20^, the present investigation demonstrated a reduction in CCT in glaucoma patients. Those affected also showed thinning of the peripheral areas of the cornea. Physiologically, the cornea thickness increases from the center to the periphery. This rise, represented by the Pentacam parameters PAC_DIFF_, was also smaller in patients compared to healthy subjects. In addition, the observation is confirmed by the parameter pachy slope, determined using the CST. This finding is consistent with the results of the population-based Gutenberg Health Study, which also found a smaller increase in peripheral corneal thickness in glaucoma^32^. To our knowledge, the present study is the first to combine Pentacam and CST measurements to examine the peripheral corneal thickness profile and associated factors in different forms of glaucoma. The only available previous report of the pachy slope in NPG patients also found significantly lower values than in healthy subjects^33^.

According to Hamilton et al. not only the central, but also the midperipheral corneal thickness influences IOP measurement^34^. The measuring head of Goldmann tonometry, which is considered the gold standard, not only affects the corneal center, but also applanates peripheral areas with its diameter of 3.06 mm^2^. Since the periphery is also thinner than in healthy people, falsely low IOP measurements are likely in glaucoma patients. In keeping with this, several previous works addressed CCT-corrected IOP measurement^35–38^. For example, according to the “Dresdner correction table” published by Kohlhaas et al., an IOP correction of 1 mmHg is required for every 25 μm deviation of the CCT from 550 μm^36^. Whitacre and colleagues recommended an IOP correction of 2 mmHg for every 100 μm CCT change^38^, and Doughty and Zaman reported a correction of 2 or 3 mmHg for a 50 μm deviation in CCT from 535 μm for eyes with chronic disease^37^. Based on previously published correction factors, the underestimation of IOP is estimated to range between approximately 0.5 and 1.5 mmHg per 25 μm reduction in CCT^36–38^. Accordingly, the mean CCT differences observed in our cohort (≈ 30 µm) may lead to an underestimation of Goldmann applanation tonometry (GAT)-measured IOP by about 1–2 mmHg. This potential bias may be slightly greater in patients, as the mid-peripheral cornea, which is also flattened by the 3.06 mm GAT prism, exhibited additional thinning. However, correction factors derived from peripheral pachymetry values are, to our knowledge, currently unavailable. Contrary to common practice, the correction values may not be directly applicable to glaucoma patients, since the original studies were conducted in healthy, elderly subjects. Although the exact correction factors may differ between healthy and glaucomatous eyes, the present findings underscore that pericentral thinning may further contribute to a systematic underestimation of IOP in clinical settings.

The differences found in peripheral pachymetry values between the forms of glaucoma are interesting. While the reduction in corneal thickness was clearly detectable in HPG and NPG, there were fewer clear deviations from the healthy group in PEXG, for example for PAC 5 mm and PAC_DIFF_ 5 mm (see Fig.1C and D). Brandt et al. previously reported that CCT varied in different forms of glaucoma in some pilot studies^39^. However, the results are inconsistent. For example, Lešták et al. compared the CCT between HPG and NPG patients and found higher values in NPG^40^. In contrast, other works showed a decrease of CCT in NPG^41,42^. In PEXG patients, a thinner CCT^41,43^ or a CCT comparable to healthy controls^44^ has been reported. There are no previous results on peripheral corneal thickness in different forms of glaucoma. The deviations between PEXG and POAG patients may be due to the different etiology of this secondary glaucoma^45^. In addition, PEXG is known to reduce the number of endothelial cells^44,46^. This could lead to changes in the corneal hydration status and possibly edematous swelling. Accordingly, Módis and colleagues reported an association between the reduction in corneal ECD and the CCT increase in diabetes mellitus^47^, and Inoue et al. found an increase in peripheral corneal edema with progression of endothelial dysfunction after penetrating keratoplasty^48^. However, clinically significant corneal swelling is not a typical manifestation of PEX. Furthermore, the smaller sample size of the PEXG group compared with HPG and NPG limits the statistical power of between-group analyses. Therefore, non-significant PEXG results should be interpreted cautiously, as they may partly reflect underpowering rather than true equivalence. To allow assessment independent of sample size, standardized effect sizes (η^2^ or r) are reported for the subgroup comparisons.

In addition, an influence of the lens status would be conceivable, since uneventful phacoemulsification was the only surgery in the past that was not an exclusion criterion, and the proportion of pseudophakic patients in the PEXG group was slightly higher than in the other groups (see Table 1). In addition to the surgery-related endothelial cell loss, the incision-related corneal scar could have an influence. A previous study consistently reported that there was a higher peripheral corneal thickness after phacoemulsification with longer duration and higher cumulative energy^49^. However, in the present study there were no significant differences between phakic and pseudophakic patients. It can therefore be assumed that the changes in the corneal thickness profile are caused by glaucoma rather than by surgery. In line with this, Hong-Kee et al. reported that CCT after phacoemulsification in glaucoma patients and healthy individuals normalized to baseline after 6 weeks^19^.

In addition, the proteoglycan distribution within the cornea could influence the thickness profile^50^. The properties of proteoglycans depend essentially on their glycosaminoglycan chains, the proportion of which varies within the cornea^45,50–52^. They influence the hydration state through different water binding properties^52–54^ and the arrangement of collagen by acting as spacers between the fibrils^50^. Therefore, alterations in proteoglycan pattern could be another reason for changes in the corneal thickness profile.

A slight trend was observed indication that the more advanced the glaucomatous damage was, the more pronounced the alterations in corneal parameters. This was supported by the observed associations with rim area and disease duration. These findings may suggest that the progression of glaucomatous damage is associated with corneal thinning. However, the relationship should be interpreted with caution, as the cross-sectional nature of the study does not allow conclusions regarding causality or directionality. Conversely, one could also assume that glaucomatous changes progress more quickly in people with thin cornea. Previous results on CCT are inconsistent. For example, Belovay et al. reported associations with HRT parameters in glaucoma^18^, whereas Nutterova et al. found no correlations with the visual field defect in NPG^55^. Overall, large studies have suggested the importance of CCT as progression factor in glaucoma^21–23^.

Particular attention should be paid to the local antiglaucoma therapy. There is previous evidence that CAI may cause corneal edema in predisposed patients^56^. However, no influence of CAIs on the central and peripheral corneal thickness values was detectable for all forms of glaucoma in the current study. In line with this, Iester et al. reported no influence of topical CAI on CCT in glaucoma patients^57^. Interestingly, in the present cross-sectional analysis, thinner corneas were observed in glaucoma patients receiving PGAs compared with those without PGA therapy. These findings should be interpreted as associational rather than causal, because the cross-sectional design and potential channeling bias—whereby eyes with more advanced disease are more likely to receive PGAs—preclude inference about drug-induced effects. However, based on the analyzed indicators of disease severity, no substantial difference between patients with and without PGA use can be assumed in the present study. Furthermore, the results are subject to limitations because most patients received multiple medications and the isolated effect of PGAs on corneal thickness could not be evaluated in the present study design. To account for potential confounding, multivariate linear models were applied. The adjusted analyses confirmed the direction of the associations between PGA use and thinner CCT, suggesting that the relationship persists even after controlling for relevant demographic and clinical variables. To address multiplicity, pairwise group comparisons were additionally corrected for multiple testing using the false discovery rate (Benjamini–Hochberg). After adjustment, key differences—particularly in CCT, pachy slope, and PAC 5 mm—remained statistically significant, supporting the robustness of the observed association. Accordingly, PGA could promote or accelerate corneal thinning, but they are not the only cause. PGAs lower intraocular pressure by increasing uveoscleral outflow. This is thought to reflect a direct effect of PGA on prostanoid receptors of ciliary muscles. Receptor activation stimulates multiple interconnected responses and these signals lead to increased biosynthesis of matrix metalloproteinases (notably MMP-1, MMP-3 and MMP-9) and decrease of tissue inhibitors of metalloproteinases (TIMP 1 and 2)^58–61^. The importance of an imbalance in MMP activity and associated tissue remodeling in the pathogenesis of glaucomatous optic neuropathy has been discusses in the past^62,63^. According to Weinreb et al. CCT reduction could be a surrogate marker for MMP activity in glaucoma^63^. However, previous results on the influence of PGA on CCT are inconsistent, with causal factors for deviation remaining unknown^18^. Belovay et al. found that those reporting a CCT decrease under topical PGA therapy had more NPG patients included^18^. In line with this, previous speculations about possible differences in MMP activity in the pathogenesis of HPG and NPG have been made^17^.

Finally, there are some limitations to be mentioned. With regard to local therapy, it was not taken into account whether the eye drop contained preservatives and whether the combination of different active ingredients had an effect. In addition, only white European were included, and known CCT differences across ethnicities^18,22^ were not considered. Furthermore, the distinction between HPG and NPG in POAG patients was made based on the maximum untreated IOP values recorded in the medical history. Since the included subjects were not treatment-native patients, these data could not be verified, and some inaccuracy in group assignment cannot be ruled out. Although the effect of phacoemulsification surgery on corneal thickness appears to be minimal (see above), it would have been desirable to ensure similarity between groups regarding lens status. Because of the observational, cross-sectional design, longitudinal evaluation is not possible.

In conclusion, a decrease in central and peripheral corneal thickness was found in different forms of glaucoma. This finding is important because exact IOP measurements form the basis of glaucoma therapy. The change in thickness from the center to the periphery was less pronounced in glaucoma than in healthy subjects. Reduction in corneal thickness was associated with structural damage, disease duration, and PGA use in patients. Overall, corneal thinning both centrally and peripherally might be part of the pathophysiological processes occurring in glaucoma.

## Supplementary Information

Below is the link to the electronic supplementary material.

Supplementary Material 1.

## Data Availability

The dataset analyzed during the current study is available from the corresponding author upon reasonable request.
