# Supplementary material for: The corneal thickness profile in different forms of glaucoma
Source: Sci Rep. 2025 Nov 7;15:38990. doi: 10.1038/s41598-025-26680-z (PMC12595040; doi:10.1038/s41598-025-26680-z)
Supplement: Supplementary file 1 — Supplementary Material 1. [file 41598_2025_26680_MOESM1_ESM.docx]

**Supplementary table: effect sizes of the comparisons of corneal parameters between different forms of glaucoma** (P-value using ^1^ ANOVA with Bonferroni correction or ^2^ Kruskal-Wallis-test, effect size ^3^ **η² for ANOVA or ^4^ rank-biserial r for non-parametric tests (HPG vs. NPG / HPG vs. PEX / NPG vs. PEX);** mean value and standard deviation, median and interquartile range 25/75; HPG: high pressure primary open angle glaucoma, NPG: normal pressure primary open angle glaucoma, PEXG: pseudoexfoliative glaucoma, CCT: central corneal thickness, TCT: thinnest corneal thickness, PAC: average peripheral pachymetry value of concentric rings around TCT (circle diameter indicated in mm), PAC_Diff_: difference of pachymetry values between TCT and concerning concentric ring; all values indicated in μm)

|  |  | **HPG** | **NPG** | **PEXG** | **P^◊^** | **effect size** |
| --- | --- | --- | --- | --- | --- | --- |
| *Scheimpfluganalyzer Corvis ST* | | | | | | |
| **CCT** |  | 536 ± 37  536 (514; 559) | 530 ± 33  526 (504; 554) | 536 ± 39  538 (509; 557) | < 0.001^1^ | 0.11^3^ |
| **Pachy Slope** |  | 31.6 ± 21.7  30.2 (23.4; 38) | 28.9 ± 9.4  29.3 (22.8; 36.4) | 29.5 ± 11.9  29.4 (21.4; 37) | < 0.001^2^ | 0.04/0.04/0.001^4^ |
| *Pentacam HR* | | | | | | |
| **TCT** |  | 526 ± 36  526 (505; 548) | 521 ± 30  523 (504; 543) | 532 ± 31  528 (511; 557) | < 0.001^1^ | 0.094^3^ |
| **PAC 2,5 mm** |  | 540 ± 35  539 (519; 561) | 535 ± 30  536 (515; 556) | 549 ± 33  542 (525; 574) | < 0.001^1^ | 0.103^3^ |
| **PAC 5 mm** |  | 577 ± 35  577 (555; 598) | 571 ± 32  572 (550; 595) | 587 ± 38  579 (559; 610) | < 0.001^1^ | 0.12^3^ |
| **PAC 7,5 mm** |  | 631 ± 56  631 (609; 660) | 628 ± 34  627 (603; 649) | 629 ± 74  641 (612; 672) | < 0.001^2^ | 0.085/0.063/0.18^4^ |
| **PAC_DIFF_ 2,5 mm** |  | 14 ± 4  14 (12; 16) | 14 ± 2  13 (12; 16) | 17 ± 15  14 (12; 17) | < 0.001^2^ | 0.06/0.05/0.11^4^ |
| **PAC_DIFF_ 5 mm** |  | 51 ± 11  51 (45; 57) | 50 ± 7  50 (45; 55) | 54 ± 26  50 (45; 59) | < 0.001^2^ | 0.033/0.011/0.041^4^ |
| **PAC_DIFF_ 7,5 mm** |  | 102 ± 53  107 (95; 122) | 106 ± 15  108 (98; 117) | 84 ± 110  107 (94; 122) | < 0.001^2^ | 0.021/0.014/0.014^4^ |
